# Matrix‐assisted diffusion‐ordered spectroscopy: choosing a matrix

**DOI:** 10.1002/mrc.4459

**Published:** 2016-06-07

**Authors:** Nilce V. Gramosa, Nágila M. S. P. Ricardo, Ralph W. Adams, Gareth A. Morris, Mathias Nilsson

**Affiliations:** ^1^ Departamento de Química Orgânica e Inorgânica, Centro de CiênciasUniversidade Federal do CearáCP 12 200CEP 60455‐970FortalezaBrazil; ^2^ School of ChemistryUniversity of ManchesterManchesterM13 9PLUK

**Keywords:** NMR, ^1^H, DOSY, matrix‐assisted DOSY, MAD, dihydroxybenzene isomers, Brij, SDS, CTAB

## Abstract

Diffusion‐ordered spectroscopy (DOSY) is an important technique for separating the NMR signals of the components in a mixture, and relies on differences in diffusion coefficient. Standard DOSY experiments therefore struggle when the components of a mixture are of similar size, and hence diffuse at similar rates. Fortunately, the diffusion coefficients of solutes can be manipulated by changing the matrix in which they diffuse, using matrix components that interact differentially with them, a technique known as matrix‐assisted DOSY. In the present investigation, we evaluate the performance of a number of new, previously used, and mixed matrices with an informative test mixture: the three positional isomers of dihydroxybenzene. The aim of this work is to present the matrix‐assisted DOSY user with information about the potential utility of a set of matrices (and combinations of matrices), including ionic and non‐ionic surfactants, complexing agents, polymers, and mixed solvents. A variety of matrices improved the diffusion resolution of the signals of the test system, with the best separation achieved by mixed micelles of sodium dodecyl sulfate and cetyl trimethylammonium bromide. The use of mixed matrices offers great potential for the analyst to tailor the matrix to a particular sample under study. © 2016 The Authors Magnetic Resonance in Chemistry Published by John Wiley & Sons, Ltd.

## Introduction

Diffusion‐ordered spectroscopy (DOSY) is a powerful NMR technique for the analysis of intact complex mixtures in solution.^1^ It works by encoding the different diffusion behavior of signals from different species in a series of pulsed field gradient spin or stimulated echoes.^2^ The end result is typically presented as a two‐dimensional spectrum, with chemical shifts displayed in one dimension and diffusion coefficients in the other. The diffusion coefficient (*D*) depends on molecular properties such as size, molecular weight and shape, as well as on solvent viscosity and temperature. It is often approximated by the Stokes–Einstein equation:

(1) $$D=\frac{kT}{6\pi \eta r_{H}}$$

where *k* is the Boltzmann constant, *T* is the absolute temperature, *r*_H_ is the hydrodynamic radius, and *η* the solvent viscosity, although in practice, the relationship between diffusion and size in solutions of small molecules is more complex.^3^

When mixture components are of significantly different sizes, DOSY can be very effective. The size difference resolvable depends critically on whether or not the signals are resolved in the NMR spectrum. When signals are resolved (the high‐resolution DOSY case), signals from species with as little as a 1% in difference in *D* can be resolved (which, other things being equal, corresponds to about a 3% difference in molecular mass given the cube root relationship between radius and volume).^4^ When signals overlap, more advanced processing methods are necessary, and one typically needs a difference of at least 30% in *D*, although in favorable cases, multivariate analysis can allow a few % to suffice.^5^, ^6^ However, in many situations, the differences in *D* between species are too small for a successful DOSY analysis – for example, in mixtures of cognate species, such as isomers – even when signals are well‐resolved in the NMR spectrum.

Fortunately, by manipulating the matrix in which such analytes diffuse, their diffusion behavior can often be altered to allow DOSY to separate the signals of species that show very similar, or even identical, diffusion in free solution.^7^, ^8^, ^9^ This has been termed matrix‐assisted DOSY (MAD) for obvious reasons. MAD, relies on differential interaction, or binding, of the analytes with the matrix and is in some ways analogous to chromatography. The method is sometimes referred to as chromatographic DOSY, particularly when using a chromatographic stationary phase as the matrix.^10^, ^11^ A change in matrix can be something as simple as a change of solvent, or a change to a mixed solvent, altering the effective hydrodynamic radii of the species of interest.^8^, ^12^ More commonly, the matrix is changed by adding a large species as a co‐solute to modulate the diffusion coefficients of the species that interact with it. Naturally, this changes the sample. Diffusion coefficients now represent a compromise between those of the bound and free analyte, giving additional information about binding affinity, but sample recovery is complicated. Important examples include micelle‐ or aggregate‐forming surfactants like sodium dodecyl sulfate (SDS) and sodium bis(2‐ethylhexyl) sulfosuccinate (AOT)^7^, ^13^; polymers like polyethyleneglycol (PEG)^14^ and polyvinylpyrrolidone (PVP)^15^; complexing agents like cyclodextrins^9^ and crown ethers^16^; and chemical shift reagents like Eu(FOD)_3_.^17^

Matrix‐assisted DOSY holds great potential for the NMR analysis of difficult mixtures such as natural product extracts, chemical reaction mixtures, and foods and beverages, and for metabolite identification. Its efficacy has been demonstrated for a number of classes of problem species, including positional isomers,^7^, ^13^ epimers,^18^ enantiomers,^16^ and natural product mixtures.^12^, ^19^ However, our understanding of how to choose a matrix for a particular type of sample is just in its infancy, and largely empirical. In this publication, we investigate the effects of various matrices on a representative mixture, illustrating the process of choosing a matrix tailored to the species under study.

**Figure 1:**
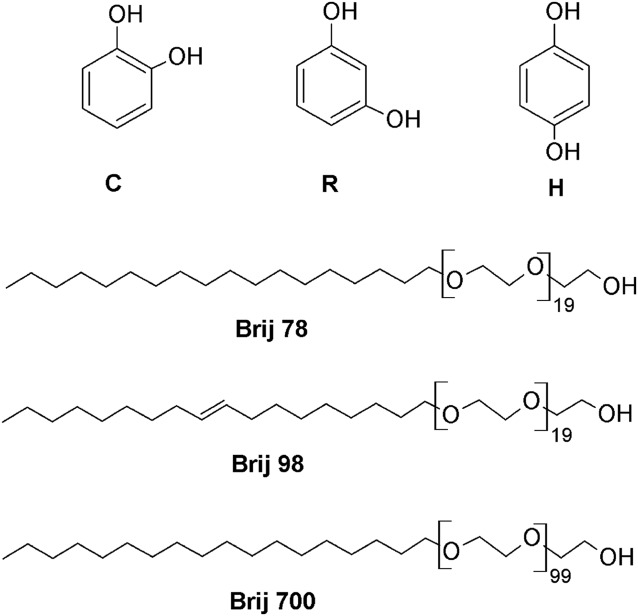
Chemical structures of catechol (C), resorcinol (R), hydroquinone (H), and the non‐ionic surfactants Brij 78, Brij 98, and Brij 700 used in this study.

Properties that affect the interaction of a solute with a matrix include its size, polarity, shape, amphiphilicity, acidity, and basicity. We have chosen to investigate the behavior of a simple, well‐characterized mixture consisting of the three positional isomers of dihydroxybenzene: catechol (1,2‐benzenediol), resorcinol (1,3‐benzenediol), and hydroquinone (1,4‐benzenediol). The chemical structures of catechol (C), resorcinol (R), and hydroquinone (H) are shown in Fig. 1, along with those of the three nonionic surfactants investigated, polyoxyethylene (20) stearyl ether (Brij 78), polyoxyethylene (20) oleyl ether (Brij 98), and polyoxyethylene glycol (100) stearyl ether (Brij 700).^19^, ^20^ The dihydroxybenzenes show very similar diffusion in aqueous solution, so the signals cannot be separated by a standard DOSY experiment. What makes them interesting in this study is the variety of properties potentially useful in MAD: subtly different shapes, determined by their substitution patterns; different polarities, reflected in their estimated water/octanol partition coefficients logP (hydroquinone 0.59; resorcinol 0.79; catechol 0.88); different degrees of amphiphilicity; and different acidities and basicities, reflected in their pKa values.^7^

We chose to study this test mixture in a selection of matrices: micelle‐forming surfactants [SDS, cetyl trimethylammonium bromide (CTAB), AOT, Brij 78, Brij 98, and Brij 700], individually and in combination; polymers (PVP and PEG); and complexing agents (*α*‐ and *β*‐cyclodextrins). The diffusion behavior was also evaluated in different solvents commonly used in NMR and in solvent mixtures.

Surfactants (normally forming micelles) are probably the most commonly used co‐solutes in MAD. Ionic surfactants, including SDS as micelles in aqueous solution and AOT as reverse micelles or aggregates in non‐aqueous solutions, have been used for signal resolution of mixtures of monomethoxyphenol isomers and of short chain alcohols,^13^, ^21^ of *cis* and *trans* isomers,^22^ and of dihydroxybenzene isomers.^7^ SDS micelles have been used in a mixed solvent, DMSO‐*d*_6_/D_2_O, for resolution of the signals of flavonoids that are poorly soluble in water.^12^ In some cases, like that of SDS in aqueous solution, the mechanism of interaction is relatively well understood, and the contributions of different factors have been quantified.^23^ The major determinant of association with micellar SDS in aqueous solution was shown to be molecular volume, which is largely uninteresting from a MAD point of view as we are only interested in manipulating the diffusion of species that have similar sizes. The next biggest contributor is hydrogen‐bond basicity, which could explain the differential interactions previously observed for the dihydroxybenzenes.

Complexing agents can form inclusion complexes by entrapping molecules of appropriate size and shape, thereby modifying their diffusion coefficients. *β*‐cyclodextrin (*β*‐CD) has been demonstrated as a matrix for resolution of the NMR signals of the epimers of naringin,^18^ while *α* and *β*‐cyclodextrins (*α*‐ and *β*‐CD) were effective for resolution of isomers of aminobenzoic acids and benzenedicarboxylic acids and of the *cis*, *trans* isomers fumaric acid, and maleic acid.^9^ Crown ethers have been demonstrated to resolve the signals of positional isomers, and chiral crown ethers to separate the signals of enantiomers.^16^

Polymers in organic solvents have been shown to be effective matrices for modulating diffusion behavior according to solute polarity. PEG was used to resolve mixtures of natural products and of steroids,^14^ and PVP for the analysis of organic mixtures.^15^

We report here on the relative efficiency of the selected matrices, including for the first time the use of mixed surfactants, in the analysis of a mixture of dihydroxybenzenes, trying to rationalize the major mode of separation for each matrix.

## Experimental

### Materials

All surfactants, cyclodextrins, polymers, and analytes were obtained from Sigma‐Aldrich and were used without further purification. Stock solutions of catechol, resorcinol, hydroquinone, AOT, Brij 78, Brij 98, Brij 700, *α*‐ and *β*‐CD, CTAB, SDS, PEG, and PVP were prepared in D_2_O and CDCl_3_ according to solubility to obtain the concentrations used in this study.

### NMR study

All NMR experiments were performed on a Varian INOVA 400 MHz spectrometer equipped with a 5 mm inverse detection z‐gradient probe capable of producing a maximum nominal gradient of 30 G cm^−1^. The Oneshot pulse sequence^24^, ^25^ was used for DOSY experiments, with gradient pulse durations (*δ*) of 2.0–6.3 ms, a diffusion delay (Δ) of 200 ms, and nominal gradient strengths ranging from 5 to 27 G cm^−1^, with 16 gradient amplitudes increased in equal steps of gradient squared. All spectra were recorded with 32 768 time domain data points. Experiments in D_2_O were carried out with temperature control set at 298 K; for samples in CDCl_3_, the experiments were performed at 294 K to reduce the effects of convection. The NMR signals of sodium 3‐(trimethylsilyl)‐1‐propanesulfonate and tetramethylsilane (TMS) were used as internal references for D_2_O and CDCl_3_ samples, respectively, and for reference deconvolution. Raw experimental data may be downloaded from DOI: 10.15127/1.298263.

### Data analysis

Nuclear magnetic resonance data were processed using the DOSY Toolbox,^5^ instrumental inconsistencies were corrected by reference deconvolution,^26^, ^27^ and the effects of non‐uniform pulsed field gradients on diffusion coefficient determination were corrected for.^28^ The error estimates used in the figures were obtained from the data fitting. The hydrodynamic radii of the surfactants were estimated using the Stokes–Einstein equation [Eqn (1)] and literature data.^29^

## Results and discussion

**Figure 2:**
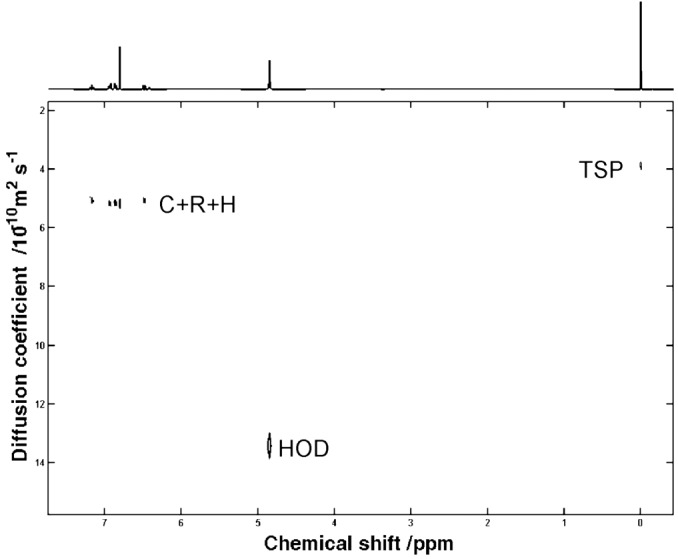
400 MHz DOSY spectrum of a sample of C, R, and H at 20 mM concentration each in D_2_O, measured at 298 K.

A standard DOSY spectrum of the test mixture of C, R, and H in D_2_O clearly shows that the signals are unresolved in the diffusion dimension (Fig. 2). It has previously been shown^7^ that by adding surfactants like SDS (in aqueous solution) or AOT (in chloroform), the mixture components show differential binding to the surfactant complexes, and hence different diffusion coefficients, allowing separation of signals in matrix‐assisted DOSY experiments.

In the first part of this work, we investigate the effect of a range of new matrices, including ionic and non‐ionic surfactants, polymers, and cyclodextrins, on the chosen test sample. The results are summarized in Table 1, showing the relative separations of signals for C, H, and R for a range of matrices, including the previously reported SDS and AOT. AOT is a special case here, as the solution is in a non‐polar solvent and is merely reported for completeness. The good general performance suggests that it is an interesting matrix for further study. It is evident that all matrices improved the separation of signals to different extents. It is worth noting that the best general separation was afforded by the nonionic surfactants Brjis 78 and 98, but that the best separation of two isomers (H from C and R) was using the positively charged CTAB (the percentage differences in diffusion coefficient between isomers were ΔCH = 49% and ΔHR = 48%). The strong interactions with CTAB correlate well with the polarities of the dihydroxybenzenes (catechol, μ = 2.62 D; resorcinol, μ = 2.07 D, and hydroquinone μ = 1.4 D).^30^

**Table 1:** Diffusion coefficients [D/10^−10^ m^2^ s^−1^] for the three positional isomers of dihydroxybenzene, catechol (C), resorcinol (R), and hydroquinone (H) in mixtures with selected matrices (M). The relative differences in diffusion coefficients of the isomers are given as ΔCR, ΔCH, and ΔHR

Matrices (conc.)^a^, [Link]	*D*_M_	*D*_C_	*D*_R_	*D*_H_	ΔCR (%)	ΔCH (%)	ΔHR (%)
DHB (20)^b^	—	5.04 ± 0.03	5.16 ± 0.02	5.18 ± 0.04	2.3	2.7	0.4
*α*‐CD (60)^c^, ^e^	2.11 ± 0.06	5.00 ± 0.008	4.81 ± 0.09	4.59 ± 0.1	3.8	8.2	4.6
*β*‐CD (60)^c^, ^e^	1.85 ± 0.01	4.74 ± 0.1	4.24 ± 0.1	4.39 ± 0.2	10.4	7.4	3.4
CTAB (100)^c^, ^e^	0.18 ± 0.003	1.87 ± 0.02	1.90 ± 0.02	3.66 ± 0.009	1.6	48.9	48.1
Brij 78 (90)^c^, ^e^	0.15 ± 0.008	1.44 ± 0.003	1.31 ± 0.005	1.95 ± 0.006	9.0	26.2	32.8
Brij 98 (80)^c^, ^e^	0.24 ± 0.001	2.17 ± 0.008	1.87 ± 0.01	2.79 ± 0.01	13.8	22.2	32.9
Brij 700 (35)^c^, ^e^	0.18 ± 0.02	3.10 ± 0.2	3.29 ± 0.2	3.52 ± 0.3	5.8	11.9	6.5
SDS (150)^c^, ^e^	0.54 ± 0.009	3.76 ± 0.09	4.16 ± 0.04	4.75 ± 0.03	9.6	20.8	12.4
AOT (200)^d^, ^e^	0.88 ± 0.03	2.54 ± 0.02	2.01 ± 0.02	3.21 ± 0.02	20.9	20.9	37.4
PVP (83.3)^c^, ^f^	0.73 ± 0.002	3.61 ± 0.04	3.13 ± 0.07	3.28 ± 0.08	13.3	9.1	4.6
PEG (166.7)^c^, ^f^	0.11 ± 0.003	2.77 ± 0.02	2.63 ± 0.01	2.96 ± 0.02	5.1	6.4	11.1

a Mixtures with catechol, resorcinol, and hydroquinone at 20 mM each in D_2_O and at 0.33 mM in CDCl_3_ (AOT).

b Mixture of C, R, and H (20 mM each) without surfactant.

^*^Solvents.

c D_2_O.

d CDCl_3_.

Concentration:

e mM.

f mg/ml.

**Figure 3:**
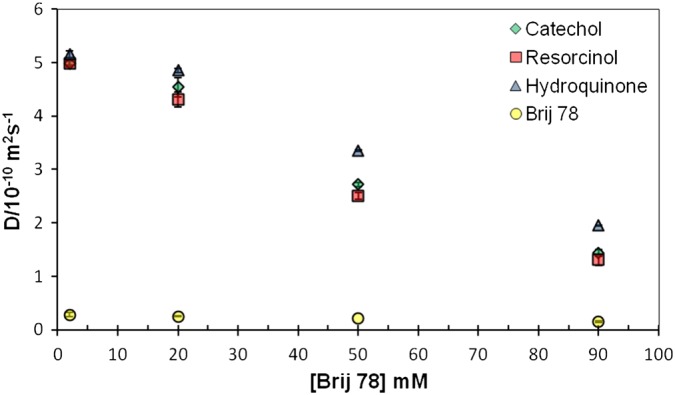
Diffusion coefficients (*D*) of C, R, and H at 20 mM concentration in samples of different Brij 78 concentrations (2, 20, 50, and 90 mM) in D_2_O at 298.

**Figure 4:**
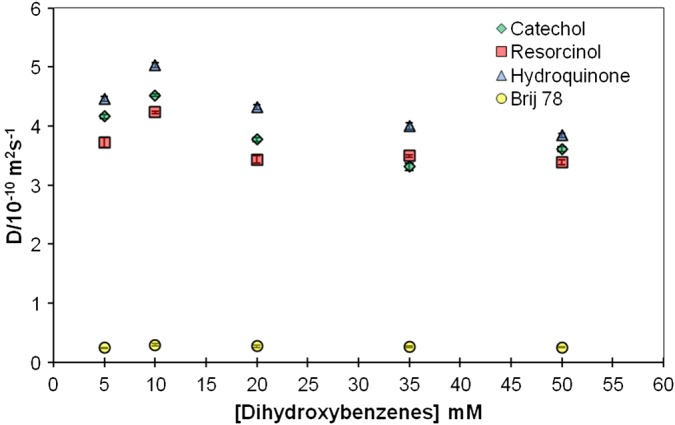
Diffusion coefficients (*D*) of C, R, and H in five samples containing 5, 10, 20, 35, and 50 mM of each isomer and 45 mM of Brij 78 in D_2_O at 298 K.

The dependence of the separation of dihydroxybenzene signals on Brij 78 concentration was investigated in more detail by measuring the diffusion coefficients of C, H, and R (at 20 mM each) as a function of surfactant concentration (Fig. 3). The concentrations of Brij 78 in all samples were above its critical micelle concentration in D_2_O (CMC = 7.6 mg dm^−3^, 25 °C).^20^ There was a general decrease in solute diffusion coefficient with concentration, reflecting a larger fraction associated with the micelles (and to a lesser extent, obstruction effects). Except for the lowest concentration (2 mM) where the solutes were in great excess, similar differences in degree of interaction between the solutes and micelles were observed. The strength of interaction increased in the order H < C < R (*p*, *o*, and *m*‐isomers) (Fig. 4).

**Figure 5:**
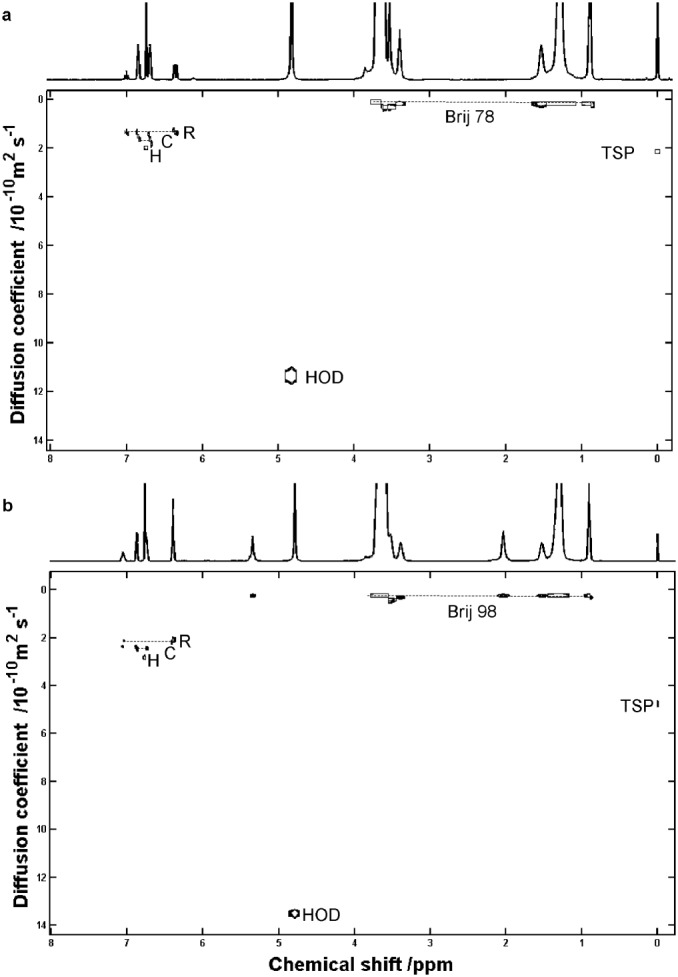
400 MHz DOSY spectra of a sample containing C, R, and H at 20 mM concentration each in D_2_O measured at 298 K with (a) Brij 78 at 90 mM and (b) Brij 98 at 80 mM.

The site of incorporation of a solute in, or binding to, a micelle depends on its structure and on the type of micelle. Polyoxyethylated non‐ionic micelles are typically arranged with their polyoxyethylene chains wrapped around a hydrophobic core formed by the hydrocarbon chains. This would suggest that apolar compounds should be located preferentially at the core of the micelle. Similar resolution of mixtures of the three isomers C, R, and H was achieved using Brij 78 at 50 mM concentration and Brij 98 at 80 mM (Fig. 5), but Brij 700 was much less effective (refer to Table 1). This may be explicable in terms of the difference in accessibility of the micellar core in Brij 700, as a consequence of its much longer ethylene oxide chains.

It is also worth noting that at higher concentrations, mixtures of solutes can have quite different behaviours because of competition between compounds for the binding site(s).^31^ A series of experiments was therefore performed in which the concentrations of C, H, and R (in an equimolar mixture) were increased for a fixed concentration of surfactant (Fig. 4). Over the whole range, good separation of signals was observed, but the separation decreased at higher proportions of solute to surfactant, with the best separation obtained at 20 mM.

Cyclodextrins have previously been reported to be effective matrices^9^, ^18^ and as expected, provided some resolution in our test system. Hydroquinone (the *p*‐isomer) showed the strongest association with *α*‐CD, reflecting the formation of an inclusion complex with cyclodextrin. This is consistent with the previous observations of Chaudhari, Srinivasa, and Suryaprakash (2013), following the sequence of interaction of different positional isomers: *p* > *m* > *o*. For *β*‐CD, the diffusion coefficients of R and H are similar, indicating that both *p*‐ and *m*‐isomers are able to fit into the cavity of the co‐solute. The binding to cyclodextrins depends on the steric interactions between solutes and the cyclodextrin cavity, with size playing a crucial role. The use of polymers as matrices in organic solvents can be very effective.^15^ However, for the aqueous test mixture used here, as expected, PVP and PEG did not show any significant differential interaction.

The variety of interaction mechanisms between solutes and the matrices suggests that it may be possible to tailor binding to suit a mixture under study by using mixtures of matrix components.^32^ In a simple‐minded approach, we would then expect behavior that is a weighted average of those of the different components. Clearly, this is a large oversimplification; components may interact with each other in unpredictable ways and form new types of complexes. Nevertheless, it is a useful starting point. From Table 1, it is clear that CTAB is very efficient at separating the signals of H from those of C and R, but that C and R are virtually unresolved in the diffusion dimension. Therefore, mixing CTAB with SDS or Brij (which give better separation of C and R signals) would be a sensible starting point. Alternatively, one could start with the matrix with the best general performance (Brij 78 in aqueous solutions) and see whether the separation of signals could be further improved by adding a second matrix component.

**Figure 6:**
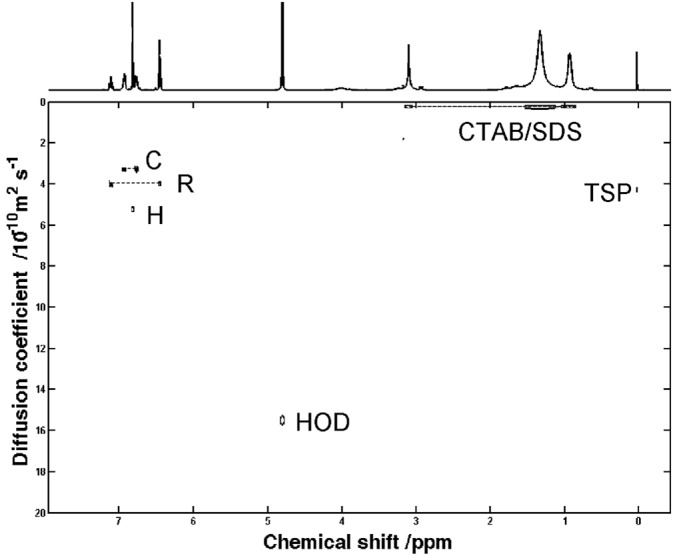
400 MHz DOSY spectrum of a sample of catechol, resorcinol, and hydroquinone at 20 mM concentration each in a sodium dodecyl sulfate (16.6 mM)/cetyl trimethylammonium bromide (6.4 mM)/D_2_O matrix.

We therefore carried out a systematic study of the surfactants used in this study using binary mixtures of Brij 78, SDS, and CTAB as aqueous matrices. The best resolution was found for a mixture of SDS and CTAB (SDS‐CTAB, *x*_CTAB_ = 0.32), with the best resolution for all three species of all the matrices investigated in this work (ΔCR 17%, ΔCH 38%, and ΔHR 25%) (Table 2, Fig. 6). Mixtures of Brijs and CTAB showed comparable separation to that of Brij 78 alone, while for the Brij/SDS mixtures, the performance was significantly worse than that of the individual matrices. We could only investigate a small range of SDS/CTAB mixtures as, at higher concentrations, highly hydrophobic mixed micelles form in a very viscous solution and CTAB precipitates.

**Table 2:** Diffusion coefficients [D/10^−10^ m^2^ s^−1^] for catechol (C), resorcinol (R), hydroquinone (H) (20 mM each), and for matrices (M) in samples of mixed surfactants: Brij 78‐SDS, Brij 78‐CTAB, and CTAB‐SDS in 0.6 ml D_2_O at 298 K

Mixed surfactants	*x* a	*D*_M_ b	*D*_C_	*D*_R_	*D*_H_	ΔCR (%)	ΔCH (%)	ΔHR (%)
Brij 78 – SDS	0.13	B: 0.24 ± 0.004	2.81 ± 0.04	2.82 ± 0.09	3.50 ± 0.03	0.4	19.7	19.4
		S: 0.32 ± 0.1						
Brij 78 – SDS	0.24	B:0.19 ± 0.008	2.85 ± 0.04	2.76 ± 0.06	3.55 ± 0.04	3.2	19.7	22.3
		S: 0.19 ± 0.06						
Brij 78 – SDS	0.5	B:0.24 ± 0.02	2.65 ± 0.04	2.75 ± 0.06	3.78 ± 0.07	3.6	29.9	27.2
		S: 0.23 ± 0.05						
Brij 78 – SDS	0.66	B:0.24 ± 0.006	2.43 ± 0.05	2.53 ± 0.08	3.68 ± 0.05	4.0	34.0	31.3
		S: 0.24 ± 0.03						
Brij 78 – CTAB	0.12	B:0.23 ± 0.01	3.54 ± 0.2	3.21 ± 0.2	4.05 ± 0.2	9.3	12.6	20.7
		C:0.40 ± 0.1						
Brij 78 – CTAB	0.22	B: 0.22 ± 0.01	3.27 ± 0.09	2.95 ± 0.03	3.88 ± 0.07	9.8	15.7	24.0
		C:0.37 ± 0.1						
Brij 78 – CTAB	0.52	B:0.22 ± 0.005	2.24 ± 0.01	2.16 ± 0.03	3.29 ± 0.06	3.6	31.9	34.3
		C:0.22 ± 0.02						
Brij 78 – CTAB	0.66	B:0.15 ± 0.003	1.53 ± 0.01	1.52 ± 0.01	2.82 ± 0.007	0.7	45.7	46.1
		C:0.23 ± 0.002						
SDS – CTAB	0.17	S:0.69 ± 0.08	5.13 ± 0.1	5.39 ± 0.1	6.11 ± 0.1	4.8	16.0	11.8
		C: 0.68 ± 0.06						
SDS – CTAB	0.32	S:0.24 ± 0.06	3.27 ± 0.01	3.93 ± 0.01	5.23 ± 0.02	16.8	37.5	24.9
		C: 0.23 ± 0.005						

a Mole fraction of the second surfactant.

b Brij 78 (B), CTAB (C), and SDS (S).

It is interesting to look at the diffusion coefficients of the mixed matrix components. In mixtures of Brij 78 and SDS, the two surfactants show very similar values, suggesting that a mixed micelle is formed, and this is also true for SDS and CTAB mixtures. However, for a mixture of Brij 78 and CTAB, the individual components have significantly different diffusion coefficients, suggesting either that separate micelles are formed for each species, or that different types of micelles with different proportions of CTAB and Brij 78 are formed.

Another type of mixed matrix is that of a mixed solvent. It has previously been shown that ethanol/water mixtures change the relative diffusion coefficients of C, R, and H.^8^ Here we investigated the use of ethanol and some common NMR solvents, methanol‐d_4_ and DMSO‐d_6_, in aqueous solutions for resolution of C, R, and H (Table 3). For the mixed‐solvents, D_2_O‐methanol‐*d*_4_ and D_2_O‐DMSO‐d_6_, no advantage was seen at either of the proportions studied (17 and 92% v/v). The best result was for the mixture D_2_O‐EtOH (92% v/v).

**Table 3:** Diffusion coefficients [D/10^−10^ m^2^ s^−1^] for catechol (C), resorcinol (R), and hydroquinone (H) (20 mM each) in samples with mixed solvents

Mixed solvents	Percentage (% v/v)^a^	*D*_C_	*D*_R_	*D*_H_	ΔCR (%)	ΔCH (%)	ΔHR (%)
D_2_O	—	5.04 ± 0.027	5.16 ± 0.024	5.18 ± 0.044	2.3	2.7	0.4
D_2_O‐EtOH	17	3.93 ± 0.02	3.83 ± 0.02	3.88 ± 0.03	2.5	1.3	1.3
D_2_O‐CD_3_OD	17	4.73 ± 0.03	4.63 ± 0.04	4.70 ± 0.03	2.1	0.6	1.5
D_2_O‐DMSO‐d_6_	17	3.91 ± 0.02	3.87 ± 0.01	3.96 ± 0.01	1.0	1.3	2.3
D_2_O‐EtOH	92	4.63 ± 0.05	3.50 ± 0.1	3.97 ± 0.1	24.4	14.3	11.8
D_2_O‐CD_3_OD	92	7.66 ± 0.03	6.89 ± 0.02	6.89 ± 0.03	10.1	10.1	0.0
D_2_O‐DMSO‐d_6_	92	2.00 ± 0.03	1.92 ± 0.04	2.04 ± 0.04	4.0	2.0	5.9

a Percentage of the second solvent.

It should be noted that common reference materials such as 3‐(trimethylsilyl)‐1‐propanesulfonate can themselves interact significantly with matrix components, so their signals should not be used for diffusion calibration.

## Conclusions

A range of surfactants and other co‐solutes, with different properties, are available for use in matrices for matrix‐assisted DOSY. The choice of matrix is dependent on the problem at hand. The results here show that empirical mixing of different surfactants can be used to tailor the matrix to give optimum performance for the mixture under study. It is clear that, especially for these mixed systems, we lack a clear understanding of all the underlying mechanisms, and further study would be desirable.
